# Psychological Distress among Students in Higher Education: One Year after the Beginning of the COVID-19 Pandemic

**DOI:** 10.3390/ijerph18147445

**Published:** 2021-07-12

**Authors:** Emilie Schmits, Sarah Dekeyser, Olivier Klein, Olivier Luminet, Vincent Yzerbyt, Fabienne Glowacz

**Affiliations:** 1 Psychologie Clinique de la Délinquance, Unité de Recherche Adaptation, Résilience et Changement, Faculté de Psychologie, Logopédie et Sciences de l’Education, Université de Liège, 4000 Liège, Belgium; 2 Centre d’Etude du Comportement Social, Institut de Recherche en Sciences Psychologiques, Faculté de Psychologie et des Sciences de l’Education, Université Catholique de Louvain-la-Neuve, 1348 Louvain-la-Neuve, Belgium; sarah.dekeyser@student.uclouvain.be (S.D.); olivier.luminet@uclouvain.be (O.L.); vincent.yzerbyt@uclouvain.be (V.Y.); 3 Center for Social and Cultural Psychology, Université Libre de Bruxelles, 1050 Bruxelles, Belgium; oklein@ulb.ac.be

**Keywords:** anxiety, depression, students, high education, COVID-19

## Abstract

The COVID-19 pandemic has affected the psychological well-being of students. Several stressors (such as socioeconomic and education-related contexts) could influence mental health, as well as individual and relational dimensions. This study proposes to evaluate the predictive effect of these factors on anxiety and depressive symptoms among students in higher education one year after the beginning of the pandemic. A sample of 23,307 students (Mage = 20.89; SD = 1.96; 69.08% of women) was assessed through an online self-report questionnaire including adapted and validated measures. The main rates were as follows: 50.6% of students presented anxiety symptoms; 55.1% reported depressive symptoms; 20.8% manifested suicidal ideations; 42.4% saw their financial situation deteriorate; 39.1% felt they were dropping out of school. One year after the beginning of the pandemic, students in higher education are anxious and depressed, especially those who identify as women (for both anxiety and depression) and as a non-binary gender (only for anxiety), experience a deterioration in their financial situation, are dropping out of school, or manifest hostility (for both anxiety and depression). The degree of study affects the symptoms’ severity (Bachelor 2 and 3 for anxiety and Master for depression). Contact with family and friends (for both anxiety and depression) as well as regular physical activity (only for depression) should provide some protection against psychological distress. Policy-makers must make a long-term investment in the well-being and positive mental health of the student community.

## 1. Introduction

The impact of the COVID-19 pandemic on the mental health of young people is clear [1]. This age group is particularly at risk of experiencing psychological distress during the crisis [2]. Recent literature suggests that the pandemic has affected students’ psychological well-being and that academic and relational changes were sources of stress [3]. Across all academic levels, students report high rates of negative feelings, such as anxiety, stress, feeling overwhelmed, tiredness, and depression [4]. Lockdowns have led to psychological problems among students, including frustration, stress, and depression [5], especially among those without preexisting mental health conditions, who have become increasingly socially isolated and have shown signs of declining mental health [6].

A recent study [7] reports that, among university students in Bangladesh, 44.59% are suffering from severe anxiety and 48.41% are suffering from moderate anxiety. Moreover, the mean score is higher in the student sample than the Chinese national norm during COVID-19 pandemic [8]. Another study revealed high rates of anxiety (29.5%) and depression (31.7%) among the student population [9]. Longitudinal data point to a significant reduction in wellbeing and an increase in depression symptoms, from a baseline of 15% to over a third of the sample at lockdown [10]. Moreover, the rate of suicidal ideation and thoughts has reached 17.8% for the university population [11]. A complex relationship exists between fear, stress, anxiety, and depression symptoms [12], and this situation may even lead young people to express the need for professional help [4]. Gender difference emerged for anxiety, with female university students showing higher levels of anxiety [8] and fear of COVID-19 than men [12] and experiencing higher emotional difficulties overall [3]. Worries about one’s economic situation, daily life, academic delays, and social support during the pandemic have significantly influenced anxiety levels [7]. Moreover, depressive symptoms are more frequent in females and people reporting a lower socio-economic status [13].

Several other stressors also affect the psychological distress of students, including their academic future, task overload, interpersonal conflicts, and restrictions on enjoyable forms of social contact [3]. Furthermore, students have had to deal with online learning challenges [14]. Although a systematic review before the COVID-19 pandemic highlighted that distance education did not differ from traditional education in effectiveness and satisfaction [15], institutions were forced to adapt to online learning quickly and encountered a number of problems [16]. Online learning may well motivate students [17] and they especially appreciate its flexibility. However, a number of related concerns, such as unstable internet connections, less interaction with lecturers and friends or less discussion, means that many prefer face-to-face learning [18]. The quality of e-learning influences a learner’s satisfaction [19], but their attitude, motivation, and opportunities for training can also have a positive impact on the effectiveness of online courses [20]. Individuals from disadvantaged family backgrounds are more likely to have unsupportive attitudes toward online courses and are therefore at risk of greater psychological distress when COVID-related restrictions are in place [21]. Although teacher and student characteristics influence the behavioral intention to use and accept e-learning systems [22], it would appear that students’ achievements, engagement, and perceptions of success decreased during the pandemic overall [23].

Frustrations generated by the lockdown conditions [5] can lead to individual and relational issues. In particular, younger people and those who had an initial stressful response to the pandemic have tended to report more hostility towards the current health crisis [24]. Research [25] demonstrates an increase in aggressive behavior and the internalization and externalization of problems during the lockdown. These could affect the psychological distress of students during this particularly stressful period. At the same time, this context has challenged their ability to adapt and cope with adversity. Certain coping strategies have partially mediated the effect of stressors on psychological health [3]. Students who managed to go outside more often, exercised more, and perceived stronger social support from their family and friends seem to be more resilient [26]. The use of social media also came as an adaptive way to keep in touch with friends and family and to deal with loneliness [27]. Among students, physical activity can decrease stress and increase academic performance [28].

The present study proposes a global approach to the analysis of psychological distress among students, taking into account socioeconomic and education-related contexts, as well as individual and relational dimensions in the prediction of anxiety and depressive symptoms related to the COVID-19 pandemic.

## 2. Materials and Methods

### 2.1. Participants

In this study, we collected data from 23,307 respondents from the student population through an online self-report questionnaire. Participants were aged between 18 and 25 years (M = 20.89, SD = 1.96), 69.08% were women, 29.97% were men and 0.96% reported a non-binary gender. Among them, 80.73% were Belgian, 10.38% were French and 8.89% were from another country. Concerning marital status, 51.06% were single, 40.60% were in a long-term stable relationship, 8.06% were in an intermittent relationship and 0.28% were married. Regarding the living environment, 45.08% lived with parents and siblings, 21.13% lived with roommates, 17.74% lived only with their parents, 9.51% lived alone, 6.39% lived with a partner and 0.15% lived with a partner and children. Among the respondents, 58.47% attended a university, 37.59% attended high school, and 3.94% attended a school of arts. In the present sample, 70.5% reported no infection by the coronavirus, 15.7% had tested positive for the coronavirus, and 13.9% thought that they had been infected but had not been tested.

### 2.2. Measures

We secured the data through an online self-report questionnaire (approved by the Ethics Committee of the Faculty of Psychology of the University of Liège) distributed via institutions (and also via social media) to all higher education students during the second partial lockdown in the country. The data collection took place between 22 February and 5 March 2021. The present sample represents approximately 10% of all students enrolled in higher education in the French-speaking regions of Belgium. It is important to note that the Belgian education system is public and open to all students. All respondents answered one list of questions. In addition, we randomly assigned different additional groups of questions to the respondents. This was done so as to keep the duration of the questionnaire to an acceptable level and explains the fluctuations in the number of respondents in the analyses. Participants received no compensation for their participation.

#### 2.2.1. Sociodemographic and Economic Data

The standard sociodemographic data (such as gender, age, origin, marital status, and living environment) were collected. Information on the evolution of the participants’ financial situation during the pandemic was also collected using a 5-point Likert-type scale item, scoring from 1 “strongly deteriorated” to 5 “strongly improved” (M = 2.5; SD = 0.9; min = 1; max = 5). Coronavirus contamination was specified with three levels (not infected, infected but not tested, tested positive).

#### 2.2.2. Education-Related Variables

We examine the educational context in more detail with several variables: the type of institution (university, high school, or school of arts); the study year (first year of a bachelor’s degree, second or third year bachelor’s, master’s degree, post-master, etc.); education modalities during the pandemic (only online courses, hybrid online courses, hybrid face-to-face courses, only face-to-face courses); and investment in school and education, with a 5-point Likert-type scale item ranging from 1 “totally dropped out of school” to 5 “strongly invested” (M = 2.9; SD = 1.1; min = 1; max = 5).

#### 2.2.3. Individual and Relational Context

This study assessed various potential everyday life resilience factors, including family, social, and physical dimensions (with three separate items created). First, we proposed a two-level measure of what helped individuals manage the pandemic —contact and interaction with family (M = 3.7; SD = 1.1; min = 1; max = 5) and social networks and distant social interactions (M = 3.6; SD = 1.2; min = 1; max = 5)—using a 5-point Likert-type scale item ranging from 1 “never” to 5 “almost always”. A continuous variable assessed the number of hours per week spent doing physical activity (M = 4.9; SD = 8.1; min = 0; max = 99).

#### 2.2.4. Mental-Health Related Factors

The two subscales of the Hospital Anxiety and Depression Scale evaluated anxiety and depression [29]. The HAD is a 14-item scale that proposes seven items related to anxiety (α = 0.79; M = 10.6; SD = 4.4; min = 0; max = 21) and seven items related to depression (α = 0.74; M = 10.7; SD = 3.8; min = 0; max = 21), scoring from 0 “never” to 3 “most of the time”. Cut-off points of 8 and 11 were identified [30]. We also used a 3-item measure of hostility (argument, anger, urge to hurt), based on the Symptom Checklist-90-Revised [31,32] and scored from 0 “never” to 4 “very often” (α = 0.72; M = 3.9; SD = 2.9; min = 0; max = 12). Additionally, a single item from the SCL-90-R evaluated suicidal ideations [31,32] scoring from 0 “never” to 4 “very often” (M = 0.7; SD = 1.2; min = 0; max = 4). Finally, the protocol included a question evaluating if participants had consulted a psychologist during the crisis (Yes; No and I don’t intend to; No but I intend to).

### 2.3. Data Analysis

In this study, we used RStudio software (version 1.4.1103, 2021, RStudio, PBC, Boston, MA, USA) to perform descriptive statistics, consistency reliability, and multiple regressions. Descriptive statistics were run for all variables of interest, including suicidal ideations and consultation with a psychologist. To test our hypotheses, global predictive models were proposed: after controlling for gender, financial situation (socioeconomic data); year of education, education modalities, education investment (education-related variables); family interactions, social networks and contacts, undertaking physical activity, and hostility (individual and relational context) were considered as independent variables. Dependent variables were anxiety and depression (mental health measures). We tested two distinct models. Statistical significance was set at *p* < 0.05.

## 3. Results

### 3.1. Descriptive Statistics

Table 1 shows descriptive statistics, including financial situation, year of education, education modalities and investment, anxiety, depression, suicidal ideations and consultation with a psychologist. A total 42.4% of students saw their financial situation deteriorate and 39.1% felt they were dropping out of school. Moreover, 50.6% of respondents presented anxiety symptoms, 55.1% presented depressive symptoms, and 20.8% reported occasional to very frequent suicidal ideations. Finally, 15.2% of participants had consulted a psychologist, and 18.1% intended to do so when they responded the questionnaire.

### 3.2. Predictive Models of Anxiety and Depression

Controlling for gender, we conducted multiple regression analyses to assess the predictive role of relevant independent variables on anxiety and depression among the student sample (Table 2). With respect to anxiety, our results point to higher rates of anxiety among female and non-binary gender students, as well as those who experienced greater financial stress, were enrolled in the second or third year of a Bachelor’s degree (compared with Bachelor 1), felt more that they were dropping out of school, or reported greater hostility. Students who invested in family interaction and social networks to manage the pandemic appeared less anxious. Concerning depression, our results show higher rates of depression among female students, those who experienced greater financial stress, were enrolled in a Master’s degree (compared with Bachelor 1), felt more like they were dropping out of school or reported greater hostility. Students who invested in family interaction and social networks to manage the pandemic and who did physical activity appeared less depressed. Both models were significant.

## 4. Discussion

The present study reveals alarming levels of anxiety and depression in the student population one year after the beginning of the COVID−19 pandemic. Nearly half of the respondents saw their financial situation deteriorate and felt more that they were dropping out of school. Over a fifth reported suicidal ideations and a third expressed a need for professional help. Identifying as female, facing financial deterioration, feeling like dropping out of school, and experiencing hostility all constituted risk factors for both anxiety and depression. In contrast, investing in contact with family and social networks to manage the pandemic emerged as a protective factor. Physical activity was also a protective factor, but only against depression. Students who had already begun their studies when the pandemic hit (i.e., those enrolled in the second or third year of a Bachelor’s degree) felt more anxious, and those studying for a Master’s degree felt more depressed than students enrolled in their first year of a Bachelor’s degree. Education modalities does not seem to influence psychological distress.

Our results corroborate recent studies showing that the pandemic had a negative impact on the mental health of students, with similar or even higher rates of anxiety and depressive symptoms when compared to the general population [1,7,9]. The exacerbation of anxiety and depressive symptoms among youth during this year of crisis is worrying. A study conducted in April 2020, one month after the beginning of the lockdown, revealed that 45% of young people showed mild to severe anxiety symptoms and 56% showed mild to severe depressive symptoms [2]. The present study, using exactly the same measurement tools and thresholds, recorded rates of up to 73.4% for anxiety symptoms and 80% for depressive symptoms one year after the outbreak of the pandemic. It is therefore hardly surprising that a significant proportion of the students expressed suicidal ideations and a need for psychological help. Care strategies must be urgently developed in response to these alarming rates. Everyone must have easy access to such care, especially given that many students have witnessed a significant and stressful deterioration in their financial situation, as reported in this research. In Belgium, the authorities installed exceptional measures, such as the reimbursement of psychological help, in order to meet the needs of the population [33]. Clearly, we have to develop other forms of support for students within the educational institutions themselves (individual and group consultations, relaxation sessions, meditation, etc.).

Note also that supplementary investigations highlighted that, considering the contextual dimension, a feeling of fear associated with contracting coronavirus was present in more than half of the population and this could impact depressive symptoms, especially because of the associated medical risks. However, vaccination could counteract this fear and the related psychological distress (unpublished data). Another research article will address this relevant issue and will focus on vaccination and health measures compliance among youth.

As expected [3,8,12], women showed more anxious and depressive symptoms, and non-binary gender students clearly reported a high rate of anxiety. It is also important to note that, although the sample is predominantly female, our results reflect the Research and Higher Education Academy data [34] in terms of gender, origin, types of institutions, and years of study. In any case, the female and non-binary gender student populations require special consideration, as gender disparities have been reported in the psychological impact of the pandemic [35]. One particular study has highlighted that, worldwide, the health crisis has reduced access to gender-affirming resources and the ability to live according to their trans- and non-binary gender. This could explain a particular increase in depressive symptoms, anxiety, and suicidal ideation [36]. Flexible interventions and supporting actions are therefore required.

Students facing financial difficulties are also particularly vulnerable [7,13], which suggests that government and education institution intervention is needed to help manage academic delays and ease financial pressure in order to alleviate feelings of depression and anxiety among students [37]. Since the beginning of the crisis, the Belgian government has strengthened the social subsidies for higher education institutions. Students can benefit from direct and individualized assistance (for example, for studies, meals, rent, travel expenses, computer costs, or psychological counseling) [38]. Nevertheless, it can still be difficult for many to access and use this support. The education institutions must continue to communicate and accompany students who are going through the process of obtaining this aid, both materially, financially, and psychologically, regardless of their progress in the program.

Our results indicate that a significant number of students felt that they were dropping out of school during the pandemic [23] and that negative feeling had a significant influence on psychological distress. Academic investment and affiliation may prevent anxiety and depression in students. Surprisingly, the specific education modalities (e.g., online, face-to-face, hybrid) do not seem to play a major role in psychological distress. Certainly, previous research has focused on challenges related to online learning and its technological and relational difficulties [14,18,21], but some of these studies also indicated that students can remain motivated [17] and that they adapt relatively well to e-learning systems [22]. Still, it would appear that the preservation of face-to-face courses preserved students’ feeling of investment in their education (unpublished data). This suggests the necessity to maintain social interactions, to offer spaces for communication and to promote socialization between students as a guarantee for school engagement and maybe indirectly a support for health at this time of life.

Study level was also associated with anxiety [39], as students in the middle of their program were more anxious. This could be explained by the fact that, compared to new students, this group had experienced higher education in a crisis context for longer, increasing the potential negative impacts on their learning trajectory. Furthermore, students who were enrolled in their Master’s degree felt slightly more depressed. This could suggest a loss of hope, particularly in terms of professional prospects, as students approach the end of their studies. In fact, more severe depressive symptoms could be the result of perceived academic stress and institutional dissatisfaction, and the duration of exposure to stressors could have an impact on psychological distress [40]. Empirical work has shown that a large part of stress among students was due to their uncertainty with respect to the end of semester exams and assessments [41]. It is therefore necessary that public authorities take measures to improve the learning experience and to attenuate the negative impacts related to the COVID-19 outbreak [5]. In particular, they should encourage the deployment of innovative media that promote interaction between academic staff and students.

At an individual level, recent literature shown that the crisis context and its related stressors have exacerbated hostility and externalizing behaviors [24,25]. Our results suggest that these manifestations can themselves increase mental health difficulties, such as anxiety and depression. Moreover, the pandemic has had a particularly strong impact on the social and relational lives of students as they have been forced to cope with loneliness [27]. The continuation of contact and interaction with family and the use of social networks and media to keep in touch with friends appear to be effective strategies in combatting anxious and depressive symptoms. In the same vein, studies highlighted the fact that perceived social support protects people against anxiety [42] and depression or irritability [43]. Moreover, physical activity appears to be an effective antidepressant among the student population. It can counteract the negative effects of fear on adolescent mental health and well-being. Consequently, we should promote physical activity to support well-being [44]; for example by providing free and accessible activities, available on campus and community, thereby promoting socialization and engagement in healthy behaviors. These findings emphasize the need for monitoring and promoting mental health in university students to improve resilience in times of crisis [3].

## 5. Conclusions

In conclusion, one year after the beginning of the COVID-19 pandemic, students in higher education are anxious and depressed, especially those who identify as women (for both anxiety and depression) and non-binary gender (only for anxiety), and if they experience a deterioration in their financial situation, feel like dropping out of school, or manifest hostility (for both). Moreover, symptom severity hangs on the students’ study level (Bachelor 2 and 3 for anxiety and Master for depression). In contrast, contact with family and friends (for both anxiety and depression), as well as regular physical activity (only for depression) would seem to provide some protection against psychological distress. It is now important to manage a proper exit from this health crisis and prepare for the complete resumption of student life. In particular, one needs to anticipate the risks of decompensation following such a long period of the psychological distress. This will require appropriate training and the availability of psychological and emotional support. Additional care will also be required for those students who proved unable to access help [45]. It seems essential to mobilize, extend, implement and perpetuate in the long term, within the institutions themselves, services working for the well-being of students, reinforced coaching systems, and systematic recourse to available aid (thus promoting the destigmatization of young people in difficulty). Moreover, these support services must also be supervised and receive the appropriate resources to respond adequately to the specific needs of young people. Educational institutions must understand that they ought to make a long-term investment in the well-being and positive mental health of the student community.

## Figures and Tables

**Table 1:** Descriptive statistics.

Variable-	Modalities	%	*n*
Financial situation	Deteriorated Stable Improved	42.4 49.3 8.2	4467 5188 869
Year of education	Bachelor 1 Bachelor 2 & 3 Master Post-Master	31.7 44.3 22.7 1.2	7283 10,181 5218 281
Education modalities	Full courses online Hybrid online courses Hybrid face-to-face courses Full face-to-face courses	66.9 30 2.3 0.8	6963 3122 238 84
Education investment	School dropout Neutral School investment	39.1 31.8 29.1	4035 3291 3010
Anxiety	No symptoms Doubtful symptoms Symptoms	26.6 22.8 50.6	5884 5056 11,193
Depression	No symptoms Doubtful symptoms Symptoms	20.1 24.9 55.1	4444 5501 12,188
Suicidal ideations	Never A little Occasionally Often Very often	64.7 14.6 9.5 5.7 5.6	6744 1524 987 591 580
Psychologist	Yes No but I intend to No and I do not intend to	15.2 18.1 66.7	1575 1882 6930

**Table 2:** Multiple regression—predictive models of anxiety and depression among students.

	DV: Anxiety					DV: Depression				
	b	β	SE	T	*p*	B	β	SE	t	*p*
**(Intercept)**	11.23	0.00	0.38	29.21	<0.001	16.33	0.00	0.32	49.97	<0.001
Gender (Ref: Men) Women Non-binary gender	1.72 3.19	0.18 0.06	0.14 0.77	12.06 4.10	<0.001 <0.001	0.61 1.28	0.07 0.02	0.12 0.66	5.04 1.95	<0.001 0.05
Financial situation Year of education (Ref: Bachelor 1) Bachelor 2 and 3 Master Post-Master	−0.49 0.29 0.32 −0.13	−0.10 0.03 0.03 −0.01	0.07 0.14 0.17 0.65	−6.45 2.03 1.85 −0.21	<0.001 0.04 0.06 0.83	−0.32 0.13 0.36 −0.26	−0.07 0.01 0.04 −0.01	0.06 0.12 0.14 0.55	−5.02 1.11 2.44 −0.47	<0.001 0.26 0.01 0.63
Education modalities (Ref: Full online) Hybrid online Hybrid face-to-face Full face-to-face	−0.01 0.61 0.43	−0.01 0.02 0.01	0.14 0.41 0.64	−0.03 1.48 0.67	0.97 0.13 0.50	−0.19 −0.05 0.06	−0.02 −0.01 0.01	0.11 0.35 0.54	−1.63 −0.14 0.12	0.10 0.88 0.90
Education investment	−0.67	−0.16	0.06	−10.91	<0.001	−1.04	−0.29	0.05	−19.94	<0.001
Family interactions	−0.16	−0.04	0.06	−2.74	0.006	−0.55	−0.16	0.05	−10.77	<0.001
Social networks	−0.16	−0.04	0.05	−2.88	0.003	−0.43	−0.13	0.05	−9.08	<0.001
Physical activity	−0.01	−0.01	0.01	−0.97	0.33	−0.03	−0.08	0.01	−5.47	<0.001
Hostility	0.57	0.38	0.02	24.86	<0.001	0.36	0.27	0.02	18.32	<0.001
	R^2^ = 0.30; F = 105.1; *p* < 0.001					R^2^ = 0.33; F = 123.9; *p* < 0.001				

## Data Availability

The data presented in this study are available on request from the corresponding author. The data are not publicly available due to ethical restriction.
